# Impact of nano Fe_2_O_3_ on radiation parameters of epoxy reinforced with nano carbon

**DOI:** 10.1038/s41598-024-73139-8

**Published:** 2024-09-20

**Authors:** Mona M. Khalil, Mona M. Gouda, Mahmoud I. Abbas, Mohamed Abd-Elzaher, Ahmed M. El-Khatib

**Affiliations:** 1 Physics Department, Faculty of Science, Alexandria University, Alexandria, 21511 Egypt; 2 Department of Basic and Applied Sciences, Faculty of Engineering, Arab Academy for Science, Technology and Maritime Transport, Alexandria, Egypt

**Keywords:** Epoxy adhesive, Radiation protection, Nano carbon, N-Fe_2_O_3_, Nanoscience and technology, Physics

## Abstract

This study aims to investigate the effectiveness of iron oxide (Fe_2_O_3_) and carbon black in micro and nanoscales incorporated into an epoxy adhesive matrix for gamma-ray attenuation. The composites were prepared via a simple and cost-effective synthesis method. The grain size of powder NPs was measured using a transmission electron microscope (TEM), and the particle size was about 20 ± 5 nm and 31.46 ± 2 nm for carbon and Fe_2_O_3_, respectively. The morphological properties were characterized by a scanning electron microscope, which indicated the excellent dispersion of NPs, which blocked almost all pores of the composite and increased the capability of radiation attenuation. In addition, the chemical composition of samples using energy dispersive X-rays (EDX) and the compressive strength were measured. Furthermore, the linear and mass attenuation coefficients were determined experimentally for incident photon energies of 59.51–1408.01 keV emitted from γ-ray sources using the sodium iodide scintillation detector NaI. A comparison was conducted between the experimental data and theoretical results that obtained from XCOM software, examined the validity of the experimental results. The relation deviation rate was found to vary between 0.0008 and 2.83%. Furthermore, the measurement of the relation deviation rate between the linear attenuation coefficients of micro and nano composites revealed a range of values between 1 and 25%. Also, shielding parameters such as half-value layer (HVL), tenth-value layer (TVL), mean free path (MFP), and effective atomic number (Z_eff_) were measured. Moreover, the equivalent atomic number (Z_eq_), absorption, and exposure buildup factors for prepared samples were calculated. The results showed that the incorporation of Fe_2_O_3_ NPs enhanced the shielding capability of the composites, as evidenced by the significant reduction in gamma-ray transmission. The composite materials exhibited excellent mechanical strength, making them suitable for practical applications in radiation shielding. Furthermore, it was determined that the elevation in N-Fe_2_O_3_ concentration resulted in a direct increase in the linear attenuation coefficient, from 0.314 to 0.519 cm^−1^ at 0.5951 MeV and from 0.099 to 0.124 cm^−1^ at 0.662 MeV. Nevertheless, a slight increase was discerned in the identified mass attenuation coefficients at 0.1332 and 0.1408 MeV. The experimental data for MFP, HVL, and TVL demonstrate that the EFeC4 sample exhibits optimal performance, with values of 1.9, 1.3, and 4.4 cm at 0.5951 MeV, and at 0.661 MeV, the values are 8.04, 5.57, and 18.52 cm, while at 0.1408 MeV, the values are 12.06, 8.36, and 27.78 cm, respectively. Overall, this research highlights the potential of iron oxide-carbon/epoxy composites as efficient and reliable materials for gamma-ray protection in various fields, including nuclear power plants, medical facilities, and space exploration.

## Introduction

The radiation leakage from Japan’s first and second nuclear power plants has highlighted the persistent risks associated with nuclear energy. As contamination spreads over time, it becomes necessary for personnel to enter hazardous areas to manage the cleanup. Beyond these incidents, the expanding application of nuclear science in fields such as aerospace, industry, agriculture, military, and medicine has heightened concerns about potential radiation leaks in these areas. Consequently, there is a growing global focus on preventing and shielding against nuclear radiation^1^. Gamma radiation, the most common and ionizing form of nuclear radiation, poses significant risks to human health and living organisms. To mitigate the dangers posed by excessive exposure, various radiation-shielding composites have been developed specifically for gamma rays .

Selecting the right materials for radiation shielding involves multiple considerations. First, the type and energy of the radiation dictate the appropriate shielding material^2^. For instance, gamma rays are effectively blocked by lead, while neutrons are better shielded by concrete. The energy level of the radiation also influences the required thickness of the shielding material. Second, the extent of shielding needed is determined by the desired protection level. For example, hospitals may require thicker shielding around X-ray machines than what is necessary for laboratories dealing with radioactive sources. Lastly, the cost and availability of materials play a crucial role in material selection. Lead and its composites are commonly used for radiation shielding due to their low cost and high effectiveness in absorbing gamma rays. However, their weight and toxicity pose significant drawbacks. Cement concrete is another material frequently used in radiation shielding, although it is heavy and may have environmental impacts^3^. In addition to metals, a variety of composite materials can be utilized for radiation shielding, including PVC-hematite^4^, bentonite clay/gypsum-Bi_2_O_3_^5^, cement-bitumen^6^, glass-B_2_O_3_^7^, clay mixtures^8^, and concrete slag^9^.

In addition to the factors mentioned above, other factors that may be considered when choosing radiation shielding materials include the flexibility of the material, its ability to withstand heat and corrosion, and its impact on the environment. Conventional gamma-ray shielding composites have flexible properties are pure polymers and their composites^10^. Polymers are increasingly being explored as potential materials for radiation shielding due to their lightweight, flexible, cost-effective, non-toxic, and recyclable properties. The effectiveness of a polymer as a radiation shield is determined by factors such as density, atomic number, and the type of radiation it is intended to block. Generally, polymers with higher density and atomic number offer better protection against radiation.

Beyond pure polymers, researchers are also investigating polymer composites for radiation shielding. These composites offer several advantages over conventional shielding materials, including being lighter, more affordable, and more flexible, while retaining non-toxic and recyclable characteristics. Typically, these composites consist of a polymer matrix reinforced with high-atomic-number fillers, such as polyester-Bi_2_O_3_^11^, PMMA-Hg, Cd, and Zn^12^, PMMA-Bi_2_O_3_^13^, epoxy-C, Ni, and Bi^14^, and NBR-ZnO^15^.

Recent advancements have seen researchers enhance these polymers by incorporating nanomaterials into their matrices. Examples include HDPE-PbO^16^, silicon rubber-TiO_2_^17^, PVC-BiVO_4_^18^, slag-natural rubber^19^, and PVA-WO_3_^20^. Nanocomposites hold significant promise for radiation shielding due to the small size of nanoparticles, which allows for more effective interaction with radiation compared to bulk materials. The inclusion of nanoparticles enhances the structural and thermal properties of the materials, as well as their radiation shielding capabilities, due to improved particle dispersion and increased surface area^21^. This can result in better shielding performance, such as reduced thickness of the required shielding material.

Despite the potential benefits of nanoparticles in radiation shielding, this area of research is still relatively new, and further studies are needed to identify the most effective nanoparticles for these applications^22^. Among the various options, iron oxide nanoparticles (Fe_2_O_3_ NPs) have attracted significant interest due to their wide range of applications, including biomedicine, cosmetics, bioremediation, diagnostics, and material engineering^23^.

Fe_2_O_3_ NPs are particularly valued for their role in MRI and controlled drug release, which makes them valuable in tissue repair and tumor therapy. Their large surface area, narrow band gap, and high stability make them suitable for various critical applications. Additionally, the cost-effective biological synthesis of Fe_2_O_3_ NPs using medicinal plants has expanded the possibilities for developing novel nanomaterials with diverse applications^24^.

Iron oxide nanoparticles synthesized through environmentally friendly methods have been proven to be safe for human use, unlike those produced through chemical processes. Initial studies on the biological synthesis of Fe_2_O_3_ NPs have demonstrated their strong antimicrobial properties and notable compatibility with living organisms^25^. GM-Turkey et al.^26^ successfully fabricated high-density polyethylene/α − Fe_2_O_3_/Al nanocomposites using blend and thermal pressing techniques. The TEM images of both the α-Fe_2_O_3_ and the Al powder revealed the presence of hexagonal nanoparticles, with an average diameter of 27 nm for α-Fe_2_O_3_ and 25 nm for Al. Furthermore, it was observed that the composite exhibited enhanced γ-ray attenuation properties compared to the pure HDPE. Nanocomposites have σ_a_ and σ_e_ values in the low photon energy region higher than those of pure HDPE, as is also the case in the Z_eff_ and N_eff_ values. HDPE reinforced with Fe_2_O_3_ and Al nanofillers has a greater effect on reducing the EBF values of low and high photon energies compared to pure HDPE.

Al-Dhuhaibat^27^ conducted a study investigating the shielding measurements for epoxy samples with reinforcement materials comprising iron, lead, aluminium, and cement. It was concluded that the attenuation coefficient of the sample increases with an increase in the concentration and density of the additives, as well as a decrease in the buildup factor values. Furthermore, he indicated that this approach was effective for the use of multiple powders, and yielded satisfactory and good results in comparison to rigid materials. Tahir et al.^28^ prepared a paste comprising Fe_2_O_3_ and fly ash of varying thicknesses. The attenuation characteristics of composite specimens were investigated, revealing a correlation between attenuation coefficients and medium thickness. An inverse relationship was observed between material thickness and attenuation values; increased thickness led to lower attenuation values and reduced X-ray absorption capacity. Akyildirim et al.^29^ investigated the attenuation of fast neutrons in Fe_2_O_3_ doped with hydroxyapatite at varying concentrations. It was established that as the concentration of iron oxide increases, the attenuation of radiation also increases, resulting in a reduction in the mean free path. This work aimed to fabricate discs of a new material composite of 70% wt. of epoxy and different ratios of micro- and nano-Fe_2_O_3_-carbon. The capacity of the composite to shield gamma rays will be investigated. In addition, the mechanical, SEM, and TEM assays were identified for the prepared samples.

## Materials and methods

### Material and sample preparation

#### Materials

The carbon graphite rods, and micro-iron oxide (Fe_2_O_3_) powder were procured from a local Egyptian companies. Epoxy adhesive material that have two parts part A resin (Bisphenol-A-epichlorhydrine) and part B hardener (Polyoxypropylenediamine) were procured from a local Egyptian company. The carbon rods were crushed and finely ground to a micros size using a blender.

#### Preparation of nanomaterials

Nanomaterials were prepared by using a ball mill machine (Photon, a local company in Egypt), with a milling time of 35 h for carbon and 10 h for Fe_2_O_3_. The ratio of balls to powder is 10:1. Stainless steel balls of diameters 5, 10, and 20 mm are used as a milling medium. The milling speed was set at 600 rpm.

#### Composite preparation

First, various amounts of Fe₂O₃ and carbon black powder, either in micro or nanoscale, as indicated in Table 1, were mixed well together to break up any agglomerations. Subsequently, 70 wt% of epoxy resin was placed in a mixing container, and the metal oxide powder was gradually added while continuously stirring to ensure even dispersion. Once the metal oxide was evenly dispersed, the appropriate amount of hardener, in a 1:1 ratio with the resin, was added and thoroughly mixed. The mixture was then poured into molds with a diameter of 3 cm and a thickness of 1 cm. The composite was allowed to cure at room temperature for 24 h, followed by an additional 7 days for complete curing.

The density of each sample was determined by applying the Archimedes principle according to ASTM D 792-912433. To achieve this, a calibrated single-pan electrical balance with a precision of 0.0001 g was employed to weigh the samples and experimentally estimate volumes for the cylindrical samples^30^.

**Table 1:** Sample composite weight%.

Sample code	Density (g/cm^3^)	Epoxy wt%	Fe_2_O_3_ wt%	Carbon wt%
Micro composites				
EFeC0	1.056 ± 0.05	100	0	0
EFeC1	1.223 ± 0.05	70	10	20
EFeC2	1.297 ± 0.05	70	20	10
Nano composites				
EFeC3	1.309 ± 0.032	70	10	20
EFeC4	1.372 ± 0.032	70	20	10

#### Chemical composite analysis

**Fig. 1:**
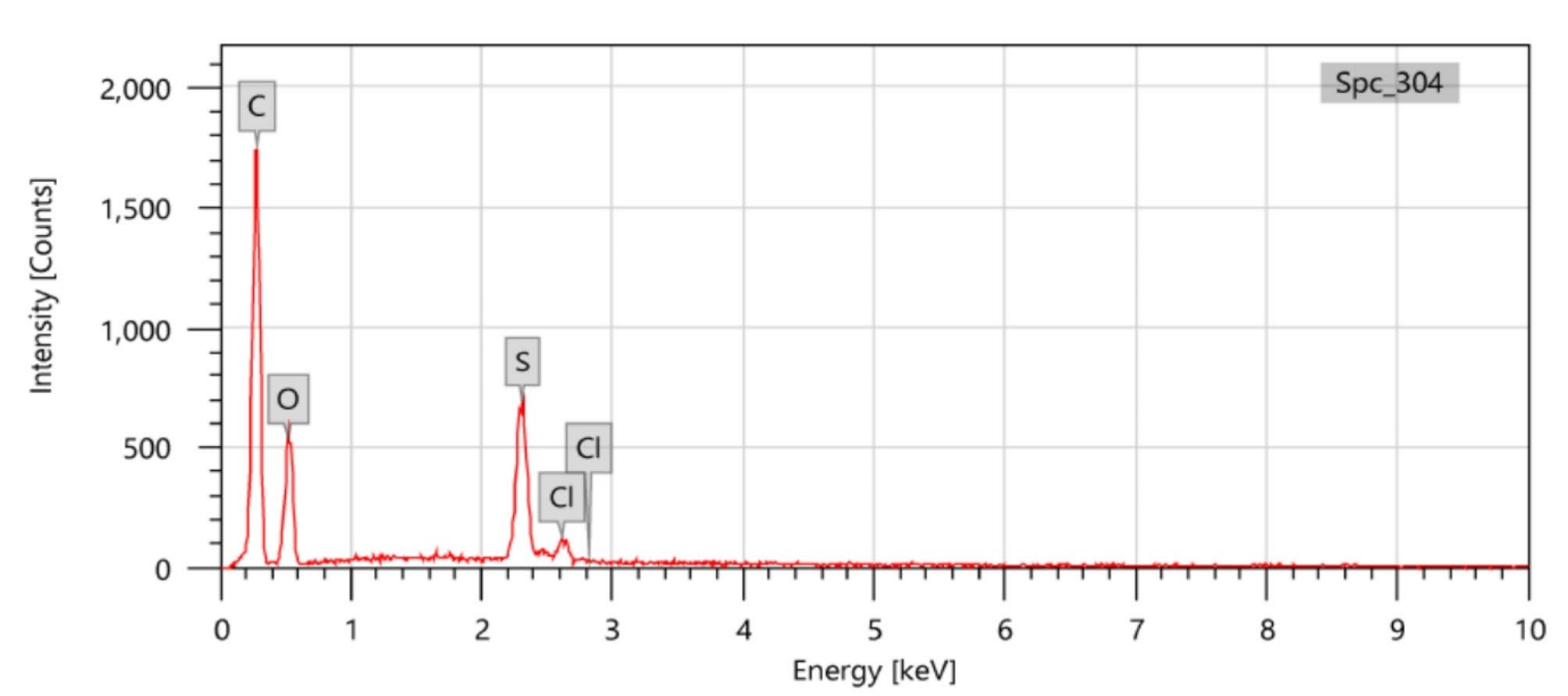
The EDX chart of Epoxy adhesive sample.

To identify the elemental composition of epoxy adhesive, the energy dispersive X-ray (EDX) measurements of JSM-5300, JEOL type, were used as shown in Fig. 1. With condition measurement, the accelerating voltage was 20 kV, the high vacuum model was used, and the magnification was ×370.

#### Morphology

##### Scanning electron microscope (SEM)

For SEM analyses, a scanning electron microscope of the JSM-5300 JEOL type was used at the Faculty of Science at Alexandria University in Egypt. For the scanning process, the samples were coated using an ion sputtering coating device, then the samples were placed inside the electron microscope with an operating voltage of 20 kV, and the magnification order was 150, 1000, and 20,000.

##### Transmission electron microscope (TEM)

TEM was performed for iron oxide (Fe_2_O_3_) and carbon NPs on a JEOL JEM-2100 high-resolution transmission electron microscope at an accelerating voltage of 20 kV at the Faculty of Science, Alexandria University, Egypt.

#### Fourier transform infrared spectroscopy

FTIR spectroscopy for all studied composites was measured at room temperature by using Bruker Tensor 37 at the Faculty of Science, Alexandria University, Egypt.

#### Mechanical measurements

Mechanical measurements were executed for pure, micro, and nano composites of area 100 mm^2^ using Devotrance Kalite Kontrol ve Test Cihazlari Ltd. equipment at Institute of Graduate Studies and Research, Alexandria University, Egypt. Measurements occur at room temperature, and the maximum load for all samples is 50 kN at a rate of 1 mm/min. Table 2 shows the loaded force for all samples.

**Table 2:** Loaded forces for all samples.

Sample’s names	Loaded force (MPa)
EFeC0	695
EFeC1	361
EFeC2	328
EFeC3	420
EFeC4	394

### Attenuation parameters

**Fig. 2:**
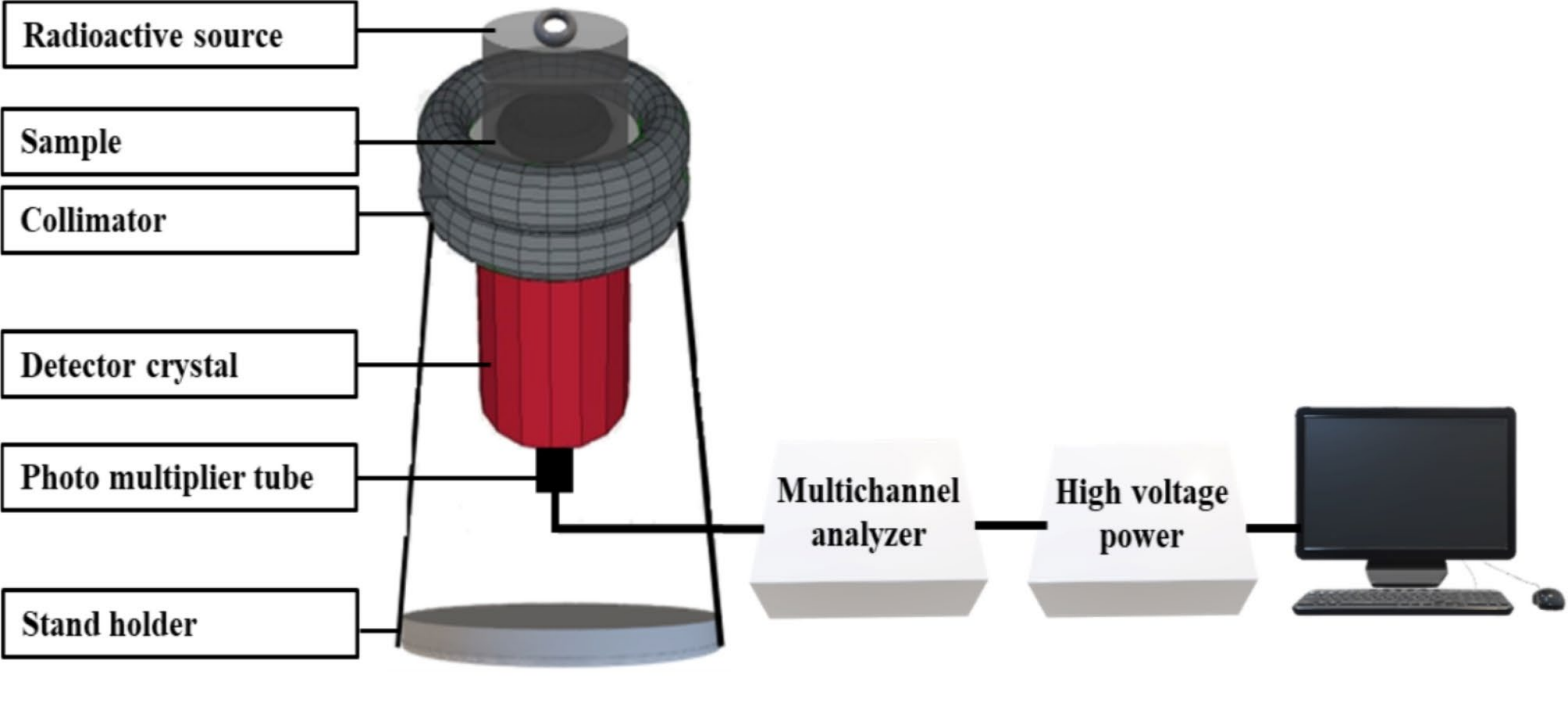
Sketching diagram of NaI(Tl) scintillation detector.

In this experiment, the shielding parameters were determined by measuring the intensity of γ-rays that passed through the specimen. The NaI (Tl) scintillation detector was employed for this purpose. Figure 2 depicts the setup configuration used in this study, with a source-detector distance of 300 mm and a detector-sample distance of 30 mm. To shield the radioactive source, a lead collimator with an outer diameter of 100 mm and an inner diameter of 8 mm was utilized as a protective shield.

The five-point radioactive sources, including Am-241, Co-60, Ba-133, Cs-137, and Eu-152, emit a wide range of energies from 0.059 to 1.408 MeV^31–33^. When these photons interact with the detector crystal, they are converted into signals that appear as peaks in a spectrum. The Genie 2000 software is utilized to analyze and interpret these signals.

To understand the material’s ability to attenuate or reduce the intensity of radiation, the area under each peak was determined simultaneously with and without the sample, yielding the transmitted intensity (I) and the initial intensity (I_0_), respectively. The first step in assessing the material’s shielding capability is to calculate the linear attenuation coefficient (LAC) using Beer-Lambert’s law^34^.

1 $$\:LAC=\frac{1}{t}{ln}\left(\frac{{I}_{0}}{I}\right)$$

In addition, the mass attenuation coefficient (MAC) was computed by dividing the estimated linear attenuation coefficient (µ) of a specific composite by its density (ρ). Furthermore, the mass attenuation coefficient (MAC) can also be calculated using Eq. 2. This calculation allows for the determination of how well the composite materials can shield against gamma radiation, taking into account their linear attenuation coefficient and density^35^.

2 $$\:{\left(\frac{\mu\:}{\rho\:}\right)}_{i}=\:{\sum\:}_{i}{w}_{i}\:{\left(\frac{\mu\:}{\rho\:}\right)}_{i}$$

where (µ/ρ) _i_ and w_i_ are the mass attenuation coefficient and the weight fraction of the ith constituent element in the composite sample, respectively. Moreover, the mass attenuation coefficient can be evaluated theoretically by employing the X-COM software code. The relation deviation of MAC results (Δ%) between experimental and theoretical results, as well as (δ%) between the micro and nano measured results, can be measured by the following equations^36^:

3 $$\:\varDelta\:\%=\frac{{MAC}_{EXP}\:-{MAC}_{XCOM}\:}{{MAC}_{XCOM}}\times\:\:100$$

4 $$\:\delta\:\:\%=\:\frac{{MAC}_{Nano}-\:{MAC}_{Micro}}{{MAC}_{Micro}}\:\times\:100$$

The typical distance traveled by incident photons between successive interactions within a medium is known as the mean-free path. This quantity can be expressed mathematically as the inverse of the linear attenuation coefficient as follows^37^:

5 $$\:MFP=\frac{1}{LAC}$$

The half-value layer (HVL) and tenth-value layer are important parameters for developing effective radiation protection materials. These values represent the thickness of the material needed to reduce the incident radiation on the substance by 50% and 10%, respectively. The HVL is determined using Eq. 6, while Eq. 7. is used to calculate the tenth value layer. These calculations provide essential information for designing materials that can effectively attenuate radiation and ensure proper shielding^38^.

6 $$\:HVL=\frac{{ln}2}{LAC}$$

7 $$\:TVL=\frac{{ln}10}{LAC}$$

The influence of the chemical composition of a photon shield is always clarified by means of the effective atomic number, Z_eff_. Additionally, the effective atomic number (Z_eff_) is used to understand photon absorption processes in different shields, enabling researchers to evaluate the relative changes in absorption with energy. Z_eff_ was calculated from (/) based on Eq. 8^39^. :

8 $$\:{Z}_{eff}=\frac{{\sum\:}_{i}{w}_{i}\:{A}_{i}\:{\left(\frac{\mu\:}{\rho\:}\right)}_{i}}{{\sum\:}_{i}{w}_{i}\:\frac{{A}_{i}}{{z}_{i}}\:{\left(\frac{\mu\:}{\rho\:}\right)}_{i}}$$

The number of electrons present per unit mass of the material is defined as the effective electron density (N_eff_). It can be used to characterize the interaction of photons with matter in radiation protection, determine the effectiveness of a material in shielding against radiation, and design more efficient shielding materials to minimize radiation exposure. N_eff_ is a function of Z_eff_ and can be calculated by using the following equation^40^:

9 $$\:{N}_{eff}=\frac{{N}_{A}{Z}_{eff}}{<A>}$$

Where < A > and N_A_ are the mean atomic mass of the sample and Avogadro’s number, respectively.

The primary element employed in the development of radiation shielding materials is referred to as the buildup factor (BF), which is divided into two categories: the energy absorption buildup factor (EABF) and the exposure buildup factor (EBF). The buildup factor is calculated using the Geometrical Progression (G-P) fitting method, which involves computations across a range of energy levels spanning from 0.015 to 15 MeV Eqs. 13 and 14. This methodology allows for a comprehensive assessment of the buildup factor, enabling the accurate design and evaluation of radiation shielding materials.

The equivalent atomic number (Z_eq_) is a key to understanding the concept of radiation shielding design. This value must fall within a specific energy range between the atomic numbers Z_1_ and Z_2_ (where Z_1_ < Z_eq_ < Z_2_), and it is calculated using the following equation^41^:

10 $$\:{Z}_{eq}=\frac{{Z}_{1}\left(log{R}_{2}-logR\right)+{Z}_{2}(logR-log{R}_{1})}{log{R}_{2}-log{R}_{1}}$$

where Z_1_ and Z_2_ are atomic numbers of elements according to ratios R_1_ and R_2_, respectively. R is the ratio of MAC _Compton_ to MAC _total_ for the sample at the same energy.

To determine the EBFs accurately, the Z_eq_ values for the investigated materials and the interpolated GP-fitting EBFs (b, c, a, X_K_, d) in the energy range of 0.015–15 MeV must be calculated using the provided interpolation formula. This calculation is essential for obtaining precise information about the materials’ behavior and properties in different energy ranges. Ensure that the interpolation process is carried out meticulously to obtain reliable results^42^.

11 $$\:C=\frac{{C}_{1}\left(log{Z}_{2}-log{Z}_{eq}\right)+{C}_{2}(log{Z}_{eq}-log{Z}_{1})}{log{Z}_{2}-log{Z}_{1}}\:$$

The GP fitting parameters C_1_ and C_2_, which correspond to Z_1_ and Z_2_ of the selected material, are taken from the ANSI/ANS-6.4.3 standard database^43^. Lastly, the EBF for the selected samples is estimated using the obtained GP fitting parameters.:

12 $$\:B\left(E,x\right)=1+\left(b-1\right)\frac{\left({K}^{x}-1\right)}{\left(K-1\right)}\:\:\:\:\:\:\:\:\:\:K\ne\:1$$

13 $$\:B\left(E,x\right)=1+\left(b-1\right)x\:\:\:\:\:\:K=1$$

where K(E, x), photon dose multiplication factor, b, build-up factor corresponding to 1 MFP which obtained by:

14 $$\:K\left(E,x\right)=C{x}^{a}+d\frac{tanh\left(\frac{x}{{X}_{k}}-2\right)-tanh(-2)}{1-tanh(-2)}\:\:\:\:\:\:x\le\:40\:mfp$$

## Result and discussion

### Morphology and structural analysis

#### Transmission electron microscopy

**Fig. 3:**
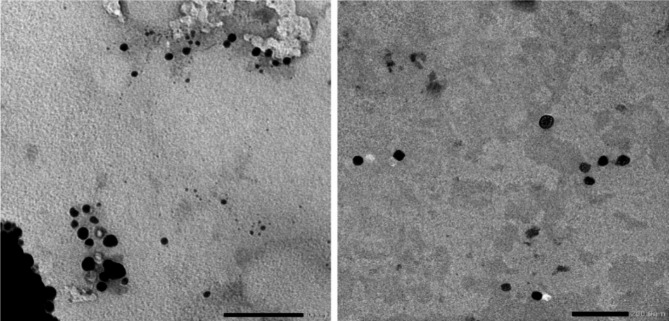
Tem images for (**a**) carbon and (**b**) Fe_2_O_3_ nano particle.

As demonstrated in Fig. 3, both iron oxide and carbon exhibit a spherical morphology, with average grain sizes of (31.46 ± 2 nm) and (20 ± 5 nm) respectively.

#### Morphological analysis

**Fig. 4:**
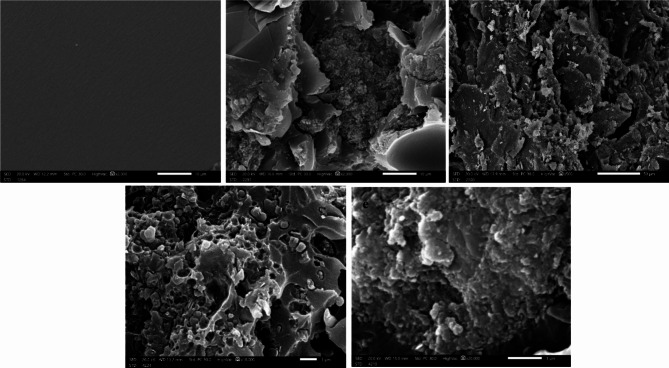
Structural SEM images EFeC0, EFeC1, EFeC2, EFeC3 and EFeC4 sample.

The microstructures of the epoxy, micro, and nano composites are displayed in Fig. 4. The scanned image of EFeC0 depicts the interface structure of the epoxy adhesive surface in its pure form; as illustrated, the surface is entirely smooth. On the other hand, the EFeC1 picture shows the presence of carbon and iron, where carbon comprises 20% of the surface while iron only accounts for 10%. Altering the percentages changes the appearance of the iron element in the image^44^.

Similarly, the EFeC3 image indicates that the percentage of carbon is higher, displaying excellent distribution and superior homogeneity compared to its micro counterpart. This can be attributed to the small size of the carbon particles. In the EFeC4 image, an augmentation in the nano-iron percentage is evident, and it is conceivable that some agglomerations of the powder might have occurred. Furthermore, it is observed that both iron and carbon exhibit homogeneity with the epoxy matrix, effectively filling most of the gaps within the sample. This amplifies the sample’s absorption of gamma rays and enhances its overall properties.

### FTIR analysis

**Fig. 5:**
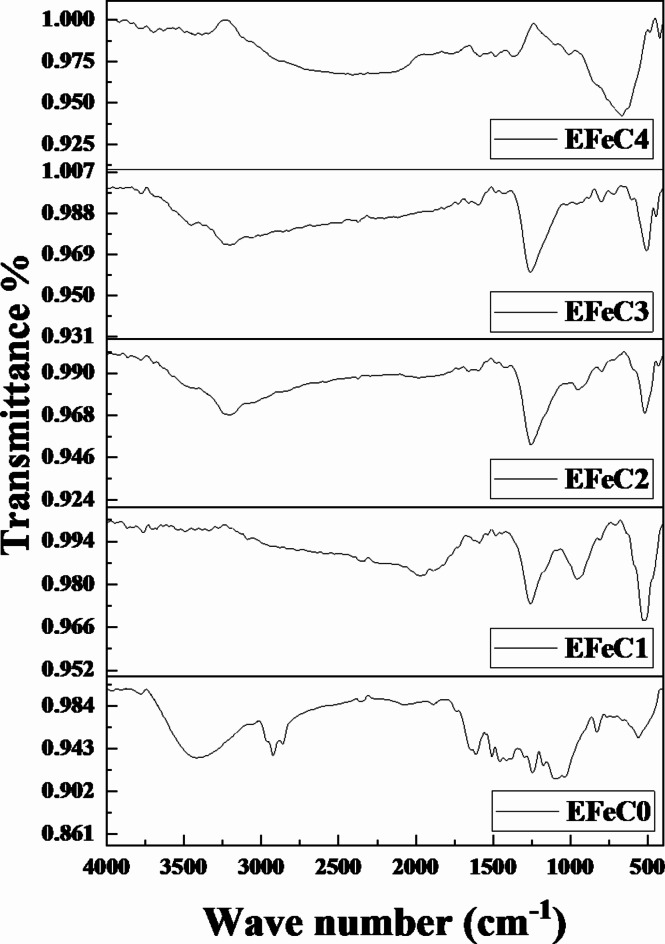
FTIR Spectrum for all studied samples.

The FTIR spectrum of the composite samples (EFeC0–EFeC1–EFeC2–EFeC3 and EFeC4) within the range of 400 to 4000 cm⁻¹ reveals several characteristic peaks. In Fig. 5 for neat epoxy, a broad peak from 3414 cm⁻¹ to 2933 cm⁻¹ corresponds to O-H stretching vibrations, indicating the presence of hydroxyl groups. A weak peak at 1888 cm⁻¹ is attributed to C=O stretching, while peaks at 1610 cm⁻¹, 1413 cm⁻¹, and 830 cm⁻¹ indicate C = C stretching and C-H bending in aromatic rings, suggesting aromatic compounds within the epoxy. A sharp peak at 540 cm⁻¹ indicates the presence of chloride monoxide. For the composite samples (EFeC1-EFeC4), a broad band due to the O-H functional group is observed from 3491 cm⁻¹ to 1924 cm⁻¹. Notably, the peak at 2923 cm⁻¹ disappears, suggesting the breaking of organic C = C bonds, which implies the dispersion of carbon and Fe₂O₃ within the epoxy. A peak at 1260 cm⁻¹ is associated with C-O stretching in esters or ethers, aligning with the presence of glycidyl ether groups in the epoxy. Finally, a strong, sharp peak at 530 cm⁻¹ indicates Fe-O stretching vibrations, confirming the presence of Fe₂O₃ in the composite^45^.

### Compression results

**Fig. 6:**
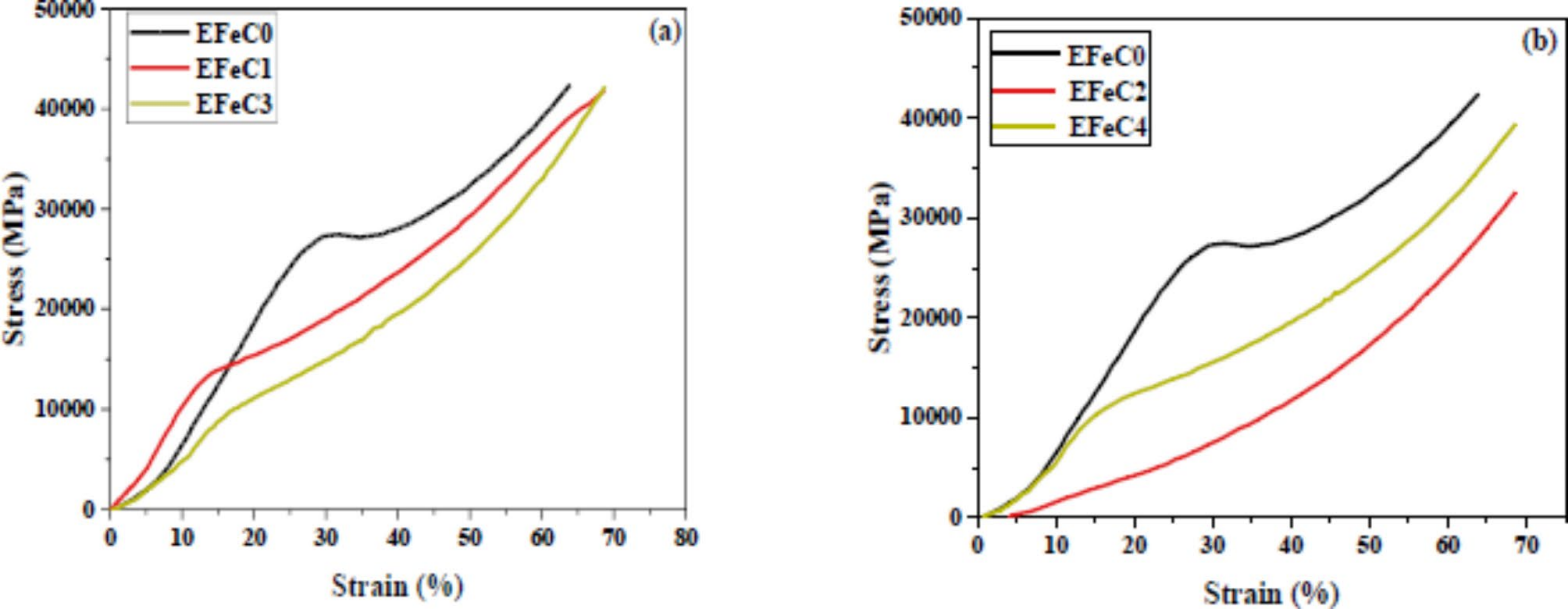
Stress–strain curves for (**a**) EFeC0, EFeC1 and EFeC3 and (**b**) EFeC2, and EFeC4.

The experimental results are illustrated in Fig. 6, which displays the compression test curves. The pure epoxy adhesive sample displayed the highest applied force, measuring at *P* = 695 MPa. The results indicated that in the sample EFeC1, the bearing strength of the epoxy adhesive decreased, leading to a reduction in the applied load to *P* = 361 MPa. Contrarily, Fig. 6a appears that the EFeC3 sample necessitated a greater applied force (*P* = 420 MPa) in comparison to the EFeC1 sample. Notably, Fig. 6b uncovers intriguing observations where the loaded force of the EFeC2 sample decreases to *P* = 328 MPa, indicating a decline in adhesive properties; however, the applied force of the EFeC4 sample increases again to *P* = 394 MPa, exhibiting an enhancement in adhesive qualities. These results indicate that the influence of any additives on epoxy adhesive is dependent on the size of the particles.

The clustering of larger microparticles in a specific region weakens the strength of the sample. Conversely, the even distribution and uniformity of nanocarbons throughout the epoxy particles enhance their mechanical properties. This is evident in the higher applied force values observed for the EFeC3 and EFeC4 samples, which increased from 361 to 420 MPa and 328 to 394 MPa, respectively. These results demonstrate the positive impact of nanocarbon on the adhesive strength of the samples^46^. The mechanical properties of the sample deteriorated as the percentage of powder increased, regardless of whether it was micro- or nanosized. This was evident in the scanning electron microscope images, which highlighted the impact of various additives on the mechanical properties of the samples. Moreover, when the percentage of nano-sized particles increased, these properties were significantly enhanced^47^.

### Attenuation analysis

The comparison between the theoretical and experimental values of mass attenuation coefficient (MAC) and the relative difference rate either between theoretical and experimental data (Δ%) or between micro and nano data (δ%) of photon energy ranges from 0.059 MeV to 1.408 MeV for the studied samples are shown in Table 3. By evaluating Δ% values using Eq. 4., it was found that there is a good agreement between experimental and theoretical values of MAC that vary between 0.0008 and 2.83%. The deviation results explained the arrangement of the experimental procedure using a narrow beam and slim absorber with the theoretical values for studying samples.

**Table 3:** Experimental and theoretical values of MAC at different photon energies and the measured densities for different composites.

Sample	Micro composite					Sample	Nano composite		
	Energy (MeV)	MAC (Exp) cm^2^/g	MAC (Xcom) cm^2^/g	Δ%	Density (ρ) g/cm^3^		MAC (Exp) cm^2^/g	δ%	Density (ρ) g/cm^3^
(EFeC0)	0.0595	0.1971	0.1950	1.0594	1.056 ±0.05				
	0.0809	0.1713	0.1739	1.4834					
	0.1218	0.1522	0.1548	1.6792					
	0.2447	0.1238	0.1244	0.4783					
	0.3443	0.1099	0.1099	0.0781					
	0.6617	0.0834	0.0839	0.6214		(EFeC0)			
	0.7789	0.0788	0.0779	1.0529					
	0.9641	0.0713	0.0704	1.2402					
	1.173	0.0649	0.0639	1.5823					
	1.333	0.0596	0.0598	0.4327					
	1.408	0.0582	0.0582	0.0190					
(EFeC1)	0.0595	0.2567	0.2545	0.8975	1.223 ±0.05		0.2975	15.8947	1.309 ±0.032
	0.0809	0.1985	0.1941	2.3079			0.2275	14.5867	
	0.1218	0.1603	0.1571	2.0530			0.1808	12.8053	
	0.2447	0.1204	0.1215	0.8298			0.1328	10.2872	
	0.3443	0.1065	0.107	0.4588			0.1164	9.3305	
	0.6617	0.0812	0.0815	0.3740		(EFeC3)	0.0880	8.4274	
	0.7789	0.0760	0.0756	0.4325			0.0815	7.2310	
	0.9641	0.0688	0.0684	0.6011			0.0721	4.7641	
	1.173	0.0621	0.0621	0.0008			0.0639	3.0519	
	1.333	0.0596	0.0581	2.4593			0.0602	1.9810	
	1.408	0.0559	0.0565	1.0306			0.0553	1.1063	
(EFeC2)	0.0595	0.3194	0.3228	1.0331	1.297 ±0.05		0.3783	25.2696	1.372 ±0.032
	0.0809	0.2193	0.22	0.3070			0.2712	23.6984	
	0.1218	0.1665	0.1638	1.6841			0.2019	21.2976	
	0.2447	0.1209	0.1218	0.6649			0.1441	19.2182	
	0.3443	0.1054	0.1068	1.2374			0.1233	16.9934	
	0.6617	0.0827	0.0811	1.9745		(EFeC4)	0.0957	15.6717	
	0.7789	0.0741	0.0753	1.6961			0.0845	14.2178	
	0.9641	0.0691	0.0681	1.4926			0.0781	13.1616	
	1.173	0.0621	0.0618	0.5605			0.0696	12.0986	
	1.333	0.0591	0.0579	2.0348			0.0656	11.1434	
	1.408	0.0579	0.0563	2.8345			0.0638	10.2555	

According to Table 3, as energy increases, the MAC values decrease. Similarly, as the percentage of Fe_2_O_3_ increases, the attenuation value also increases due to its higher atomic number. This trend can be attributed to the different interaction possibilities between photons and materials, such as the photoelectric effect, Compton scattering, and pair production. Furthermore, comparing the MAC values of micro and nanocomposites demonstrates the enhanced attenuation effectiveness of the nano size. As demonstrated in Table 3, the sample EFeC4 has a higher density than the sample EFeC2, despite having the same filler weight fraction. This discrepancy could be attributed to the decrease in dimensionality, where a reduction in particle size leads to an increase in the volume fraction of particles at interfaces or surfaces. Additionally, the discrepancy between bulk and nanocomposites was acquired in the energy region (0.059 to 1.408 MeV) and ranged from 1 to 25%. This phenomenon promotes the absorption of γ-rays and enhances the shielding properties of the material^48^.

**Fig. 7:**
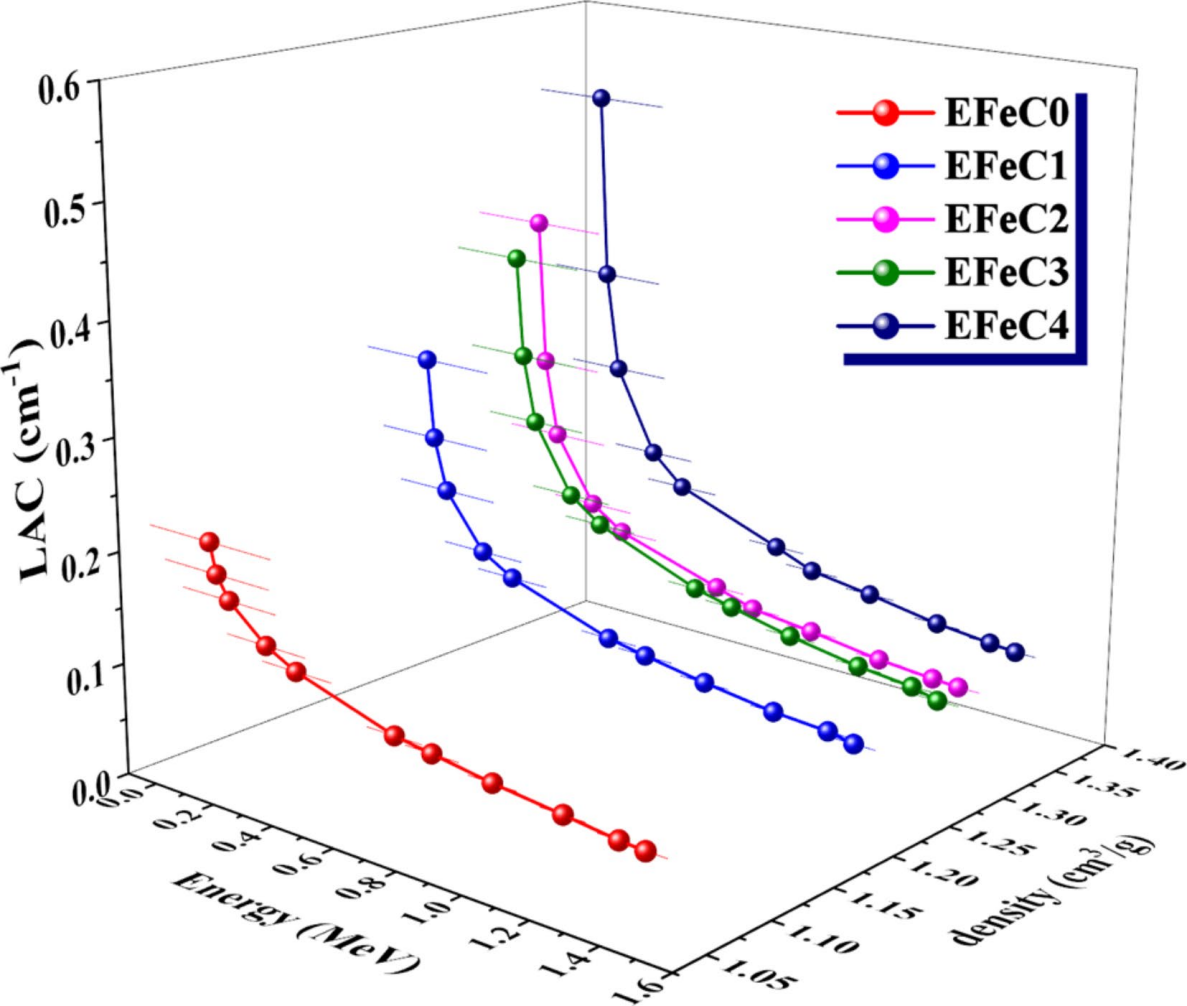
Relation of LAC with photon energy for different composites.

The efficiency of shielding materials in blocking gamma rays relies heavily on the linear attenuation coefficient (LAC), a parameter influenced by density. In Fig. 7, the relation between photon energy and the linear attenuation coefficient (LAC) is represented for various samples. The graph reveals that when energy increases, the LAC’s values decrease. Conversely, as the filler content in the composite increases, the value of the LAC increases. The LAC of the nanocomposites is found to be higher compared to the micro composites, even at the same weight%. These results can be attributed to the higher density associated with the increasing filler content.

As depicted in Fig. 7, The LAC values in cm^−1^ for EFeC1 and EFeC3 respectively, are: 0.314 and 0.386 at 0.059 MeV, 0.099 and 0.114 at 0.662 MeV, and finally 0.068 and 0.071 at 1.408 MeV. While the LAC values in cm^−1^ for 30% C micro and nano respectively, are as follows: 0.414 and 0.519 at 0.059 MeV, 0.107 and 0.124 at 0.662 MeV, and finally 0.075 and 0.082 at 1.408 MeV. This observation aligns with the expectation that higher energy gamma rays possess greater penetration properties^49^.

**Fig. 8:**
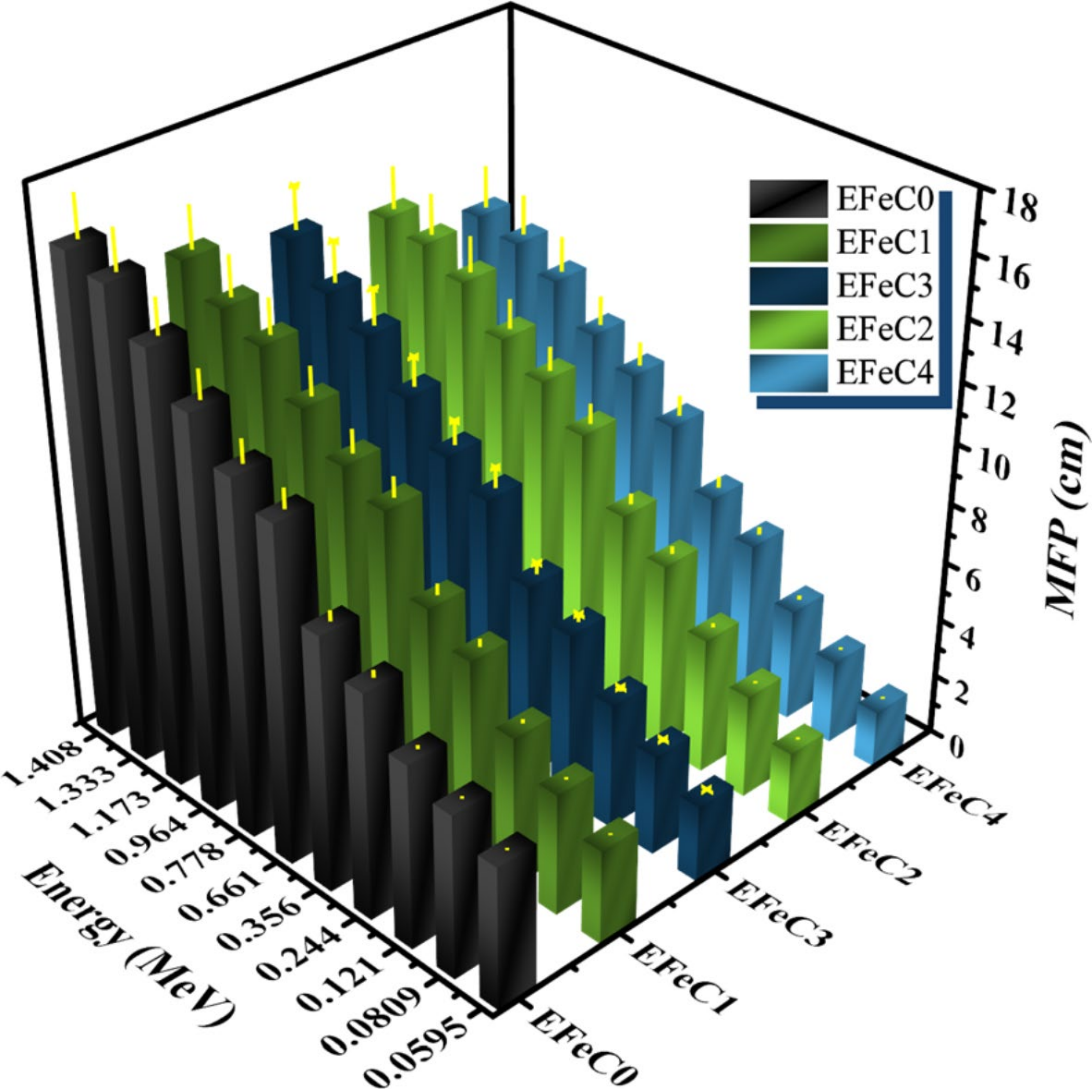
Mean free path of different weight fraction of micro and nano composites.

Figure 8 displays the plot of the mean free path (MFP) against photon energy. It is evident that as energy increases, the MFP also increases. The MFP data is arranged in the following order: EFeC0 > EFeC1 > EFeC3 > EFeC2 > EFeC4. This arrangement indicates that EFeC4 exhibits the highest radiation protection performance among the samples.

**Fig. 9:**
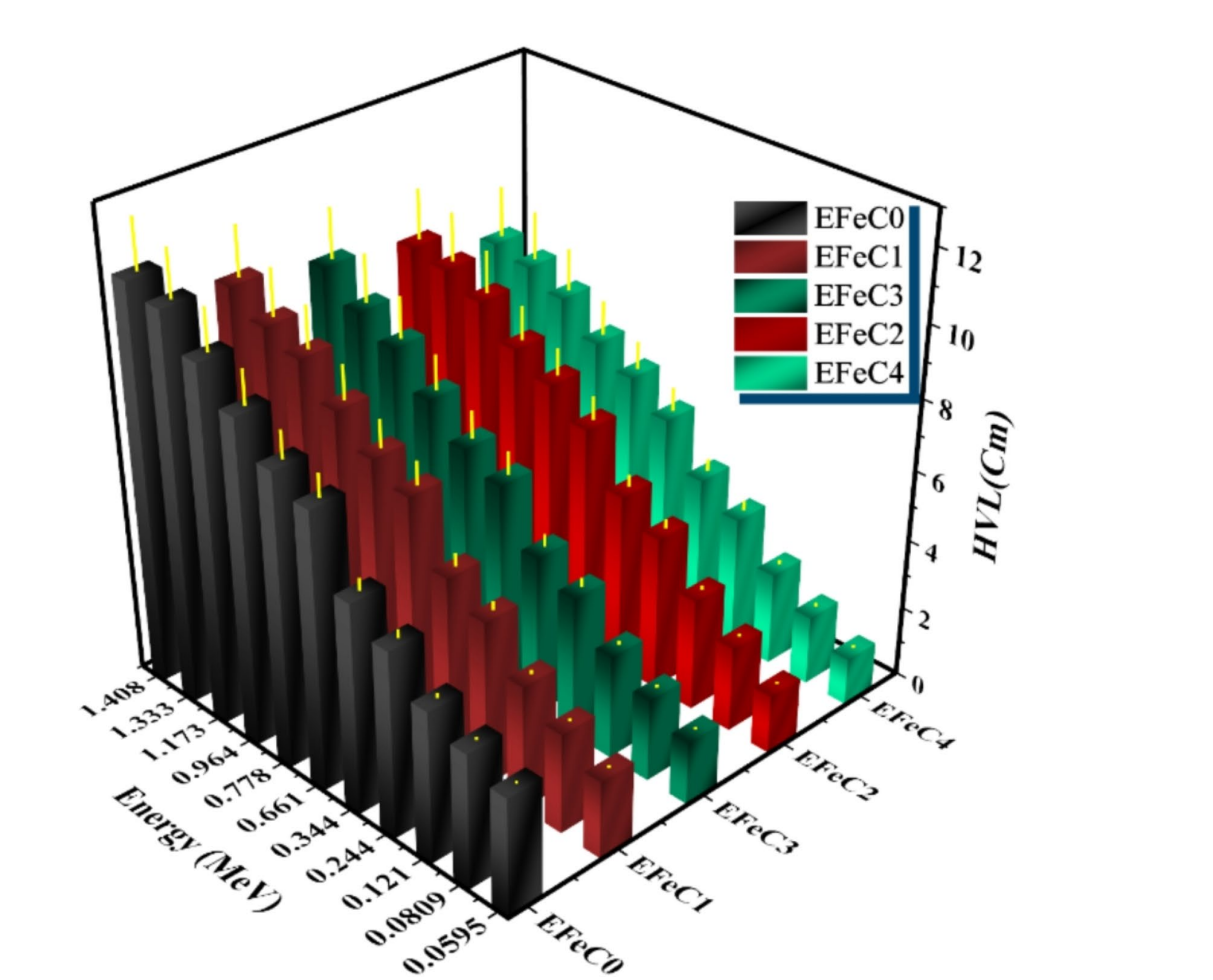
The HVL of a pure, micro and nano composite for different weight fraction at different energies.

**Fig. 10:**
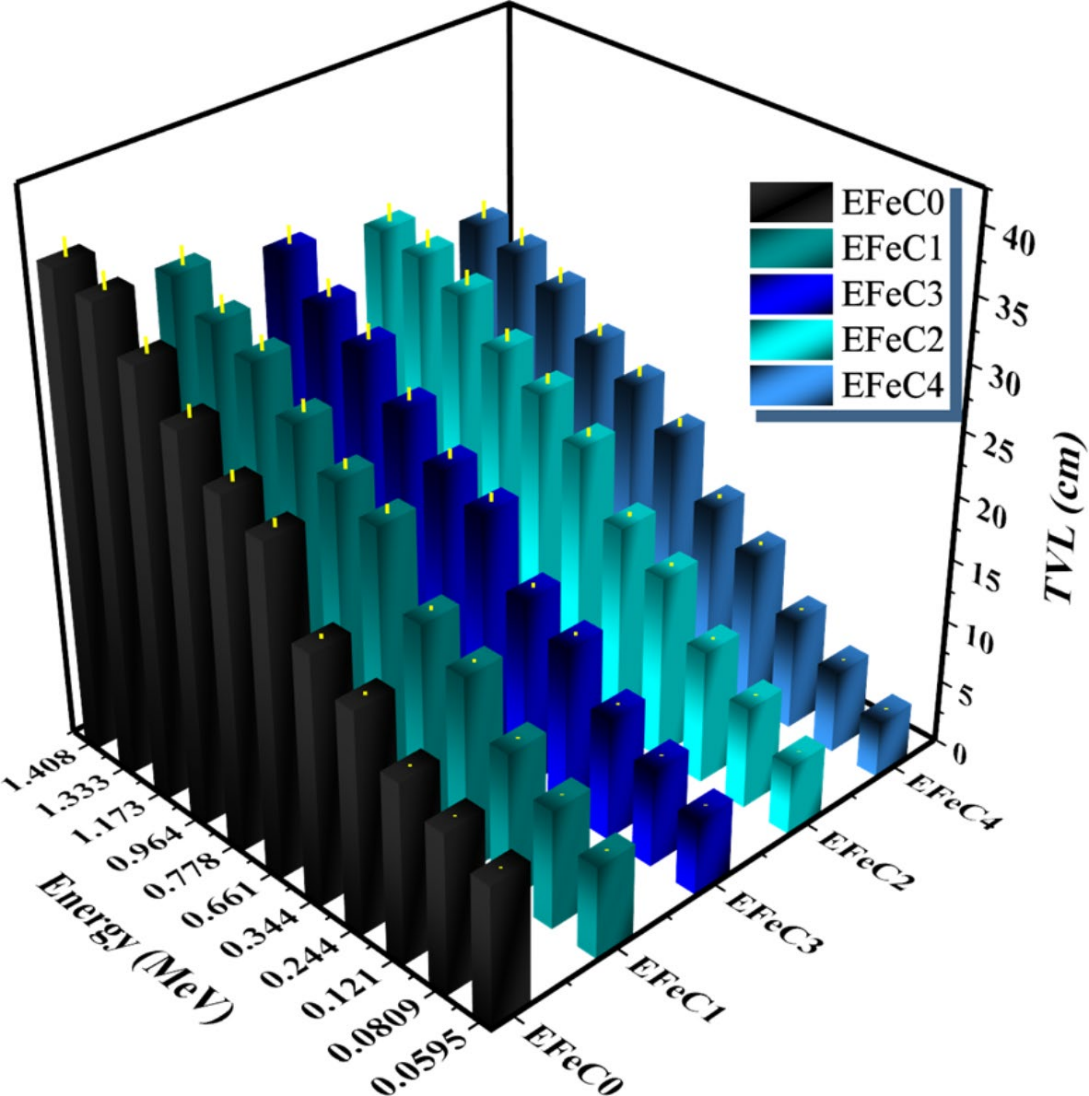
The TVL of a pure, micro and nano composites for different weight fraction at different energies.

HVL and TVL results were plotted in Figs. 9 and 10 as a function of energy. As energy increases, the values of HVL and TVL also increase. Since the HVL and TVL are shielding parameters depending on the thickness of the composite, the results indicate that HVL and TVL values of EFeC0 > EFeC1 > EFeC3 > EFeC2 > EFeC4, indicating that the best protection performance belongs to EFeC4^50^.

**Fig. 11:**
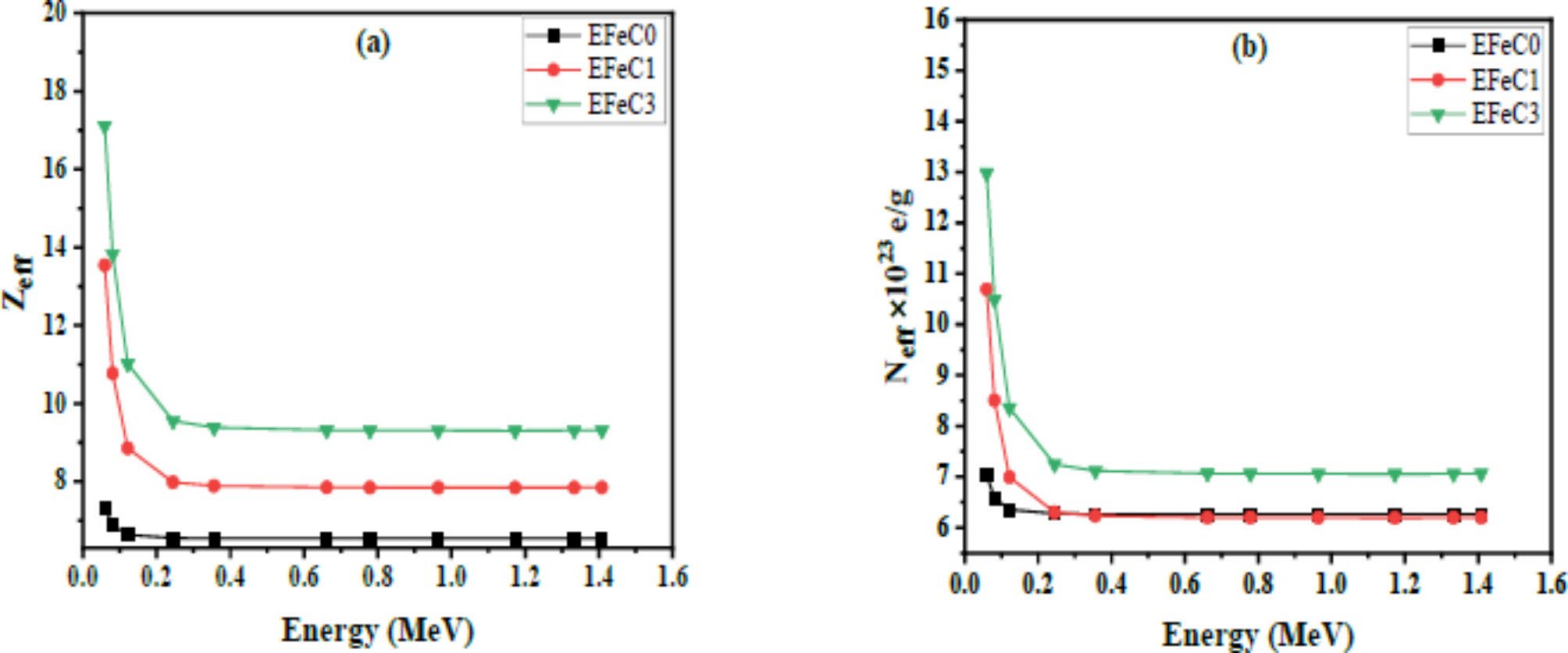
Variation of (**a**) Z_eff_ and (**b**) N_eff_ against photon energy for all samples.

Unlike elements, the atomic number of composite materials is not represented by integer values. Instead, it is denoted by the effective atomic number Z_eff_, which is a numerical quantity. Figure 11a illustrates the relationship between the effective atomic number and energy. It shows that as energy increases, the Z_eff_ values decrease. This is attributed to the dependence of the mass attenuation coefficient (MAC). Similarly, Fig. 11(b) depicts the relationship between N_eff_ values and photon energy within the range of 0.059–1.4 MeV. The behavior of N_eff_ values closely resembles the trends observed for Z_eff_ values. The N_eff_ values range from (7 to 13) electron/g at an energy of 0.059 MeV and (6.3 to 7.1) electron/g at an energy of 1.408 MeV for all the prepared samples.

**Fig. 12:**
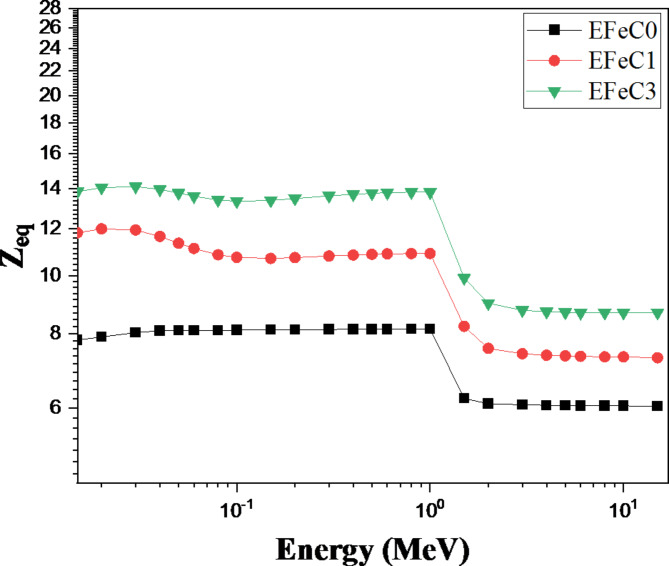
The variation between equivalent atomic number and energy for different samples.

The equivalent atomic number parameter (Z_eq_) of a material can vary depending on the incident energy. Z_eq_ is utilized to characterize a material based on its constituent elements. By calculating the ratio of the Compton cross-section to the total cross-section (MAC_sc_ / MAC_tot_), the Z_eq_ values of different materials at a specific energy can be determined^51^. In this study, the (MAC_sc_ / MAC_tot_) ratios were determined for 23 different elements (atomic numbers 4–92) across a range of energies from 0.015 to 15 MeV. The X-COM program was employed for conducting the computations, while parabolic interpolation equations were utilized to determine the Z_eq_ values^52^. The relationship between photon energy and Z_eq_ values is shown in Fig. 12, illustrating that as photon energy increases, the equivalent atomic number decreases. Additionally, increasing the concentration of carbon in the samples results in lower Z_eq_ values. Notably, at a gamma-ray energy of 1 MeV, the Z_eq_ value reaches its maximum for all samples. Beyond 2 MeV, Z_eq_ attains its minimum value. In the energy range of 0.015 to 1 MeV, Z_eq_ increases steadily due to the Compton scattering process^53^. However, beyond 1 MeV, the pair production effect becomes dominant, causing a sharp decline in Zeq with increasing energy.

**Fig. 13:**
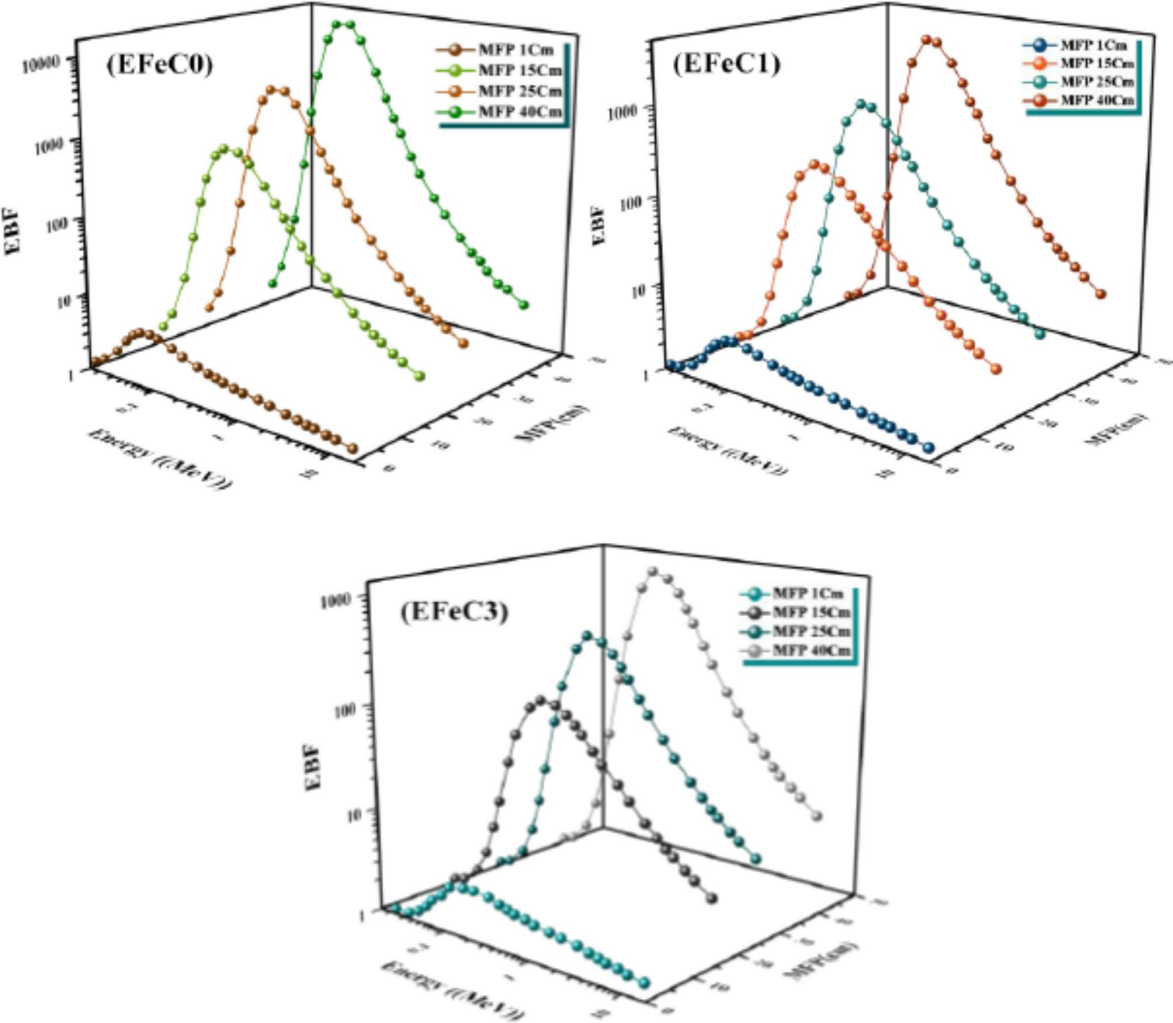
Exposure buildup factor for EFeC0, EFeC1, and EFeC2 at 1, 15, 25 and 40 mfp.

**Fig. 14:**
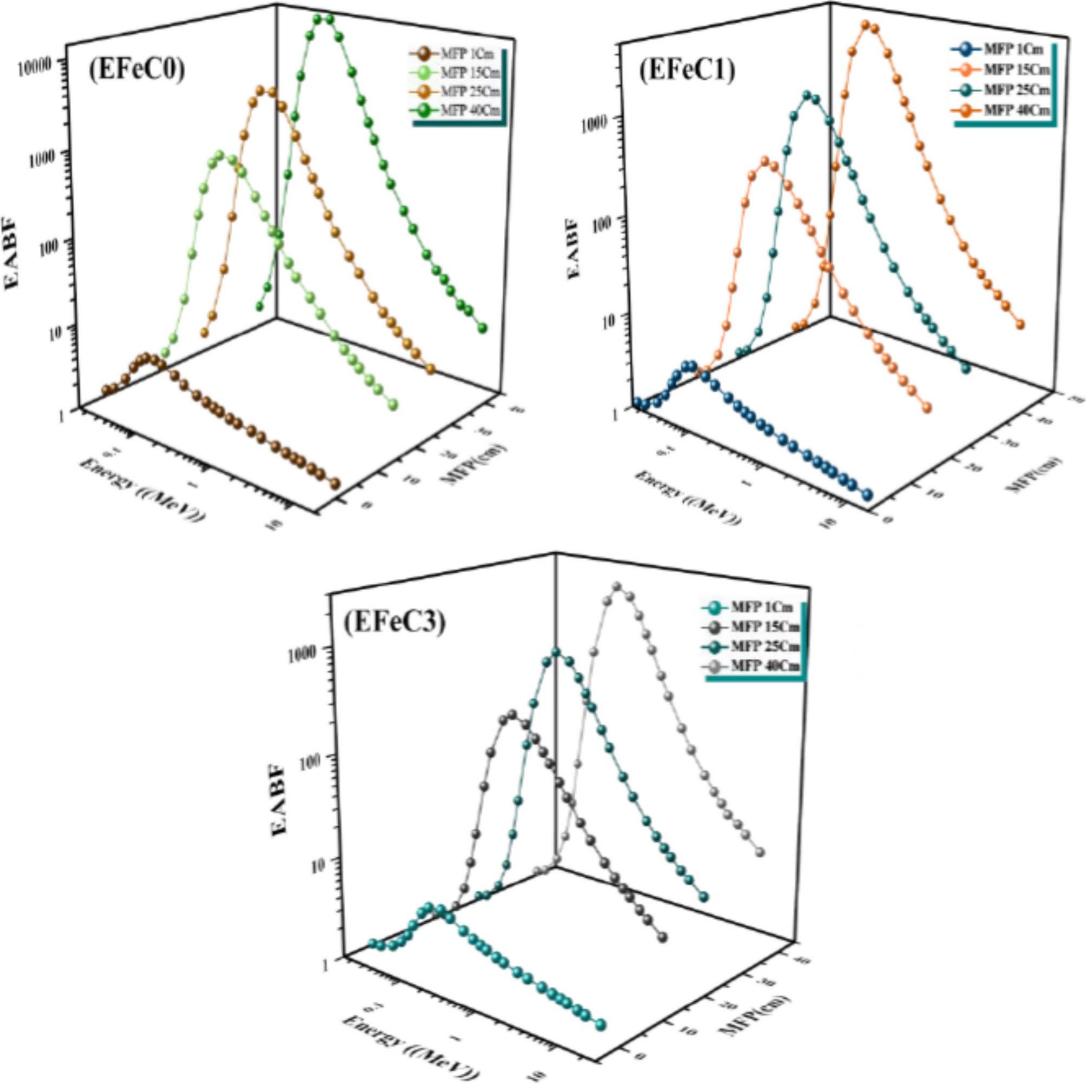
Energy absorption buildup factor for EFeC0, EFeC1, and EFeC2 at 1, 15, 25 and 40 mfp.

The buildup factors, either energy absorption buildup factor (EABF) or exposure buildup factor (EBF), for EFeC0, EFeC1, and EFeC2 at energy levels between 0.015 and 15 MeV were plotted in Figs. 13 and 14. These plots are generated for fixed penetration depth values of 1, 15, 25, and 40 MFP. The exposure buildup factors (EBFs) tend to reach minimum values at low gamma-ray energy due to the photoelectric interaction, which removes the energy of the incident photons and prevents its accumulation inside the shielding materials. Additionally, the EBF for all samples remains nearly constant at various penetration depths^54,55^.

It can be seen from Figs. 13 and 14 that the energy buildup factors (EBFs) exhibit a distinct behavior in the energy range of 0.08 to 0.2 MeV. Initially, the EBF values for all samples are small at low photon energy, as photons are primarily absorbed at this stage. However, as photon energy increases, the EBF values gradually rise until they reach a maximum peak at an intermediate energy within the specified range. This phenomenon occurs due to the dominance of Compton scattering, attributed to an increase in the number of multiple scattered photons at the intermediate photon energy, causing the higher values of the energy buildup factor in this region^56^.

Finally, at high energy levels, the exposure buildup factors (EBFs) rapidly decrease due to the absorption of photons through the process of electron-positron annihilation, leading to a prominent peak in the buildup factor values and deep penetration into the material. This phenomenon generates secondary photons. As shown in Figs. 13 and 14, increasing the penetration depth of a medium results in a corresponding increase in the thickness of the interacting medium. Furthermore, the values of the buildup factor for energies EFeC0 > EFeC1 > EFeC2 indicate that the buildup factor varies inversely with the effective atomic number (Z_eq_), as absorption processes are more dominant within materials with a higher Z_eq_ value^57^.

## Conclusion

The objective of this study was to assess the impact of incorporating different amounts of Fe_2_O_3_ and carbon, in both micro and nano sizes, into epoxy resin adhesive. The goal was to enhance the structural, mechanical, and radiation shielding properties with the aim of developing cost-effective and non-toxic materials for gamma-ray protection. Based on the results, the following conclusions can be drawn:

- The SEM images of the sample EFeC4 explained that the greater the amount of iron oxide in the composite, the better the coverage and filling of pores. It was also noted that the nano size is better in distribution and homogeneity among the composite contents.
- FTIR analysis indicates the dispersion of either carbon or iron oxide with the epoxy resin in micro and nano scale.
- The mechanical properties of the prepared samples confirmed that if the amount of metal powder increases, the compressive strength decreases, which destroys the cross-linking between the epoxy adhesive bonds and metal oxide.
- The radiation-shielding capabilities of the studied composites were analyzed in a range from 59.51 to 1408.01 keV photon energy.
- MAC and LAC values indicate that the attenuation capacity increases as the percentage of filler increases and decreases as energy increases. Also, the sample EFeC4 shows the highest attenuation capability, owing to the fact that it contains not only a higher weight fraction but also nanosized particles.
- Moreover, the values of MFP, HVL, and TVL rise in inverse proportion to the weight% of iron oxide. Also, the performance of samples containing nanoparticles is better than that of samples containing micro sized samples, so the sample EFeC4 represents the best thickness for gamma ray attenuation.
- Also, it was found that, for the samples under study, EBF values are at their highest in the region of intermediate energies, where Compton scattering is the predominant interaction. While EBF values are highest for sample EFeC0, Z_eq_ values for the EFeC2 sample are lowest, while EBF values are lowest for sample EFeC0. According to the findings, epoxy that has a high nano iron oxide content (EFeC4) has good attenuation qualities.

## Data Availability

All data generated or analyzed during this study are included in this published article.
